# Radiofrequency ablation and chemotherapy versus chemotherapy alone for locally advanced pancreatic cancer (PELICAN): study protocol for a randomized controlled trial

**DOI:** 10.1186/s13063-021-05248-y

**Published:** 2021-04-29

**Authors:** M. S. Walma, S. J. Rombouts, L. J. H. Brada, I. H. Borel Rinkes, K. Bosscha, R. C. Bruijnen, O. R. Busch, G. J. Creemers, F. Daams, R. M. van Dam, O. M. van Delden, S. Festen, P. Ghorbani, D. J. de Groot, J. W. B. de Groot, N. Haj Mohammad, R. van Hillegersberg, I. H. de Hingh, M. D’Hondt, E. D. Kerver, M. S. van Leeuwen, M. S. Liem, K. P. van Lienden, M. Los, V. E. de Meijer, M. R. Meijerink, L. J. Mekenkamp, C. Y. Nio, I. Oulad Abdennabi, E. Pando, G. A. Patijn, M. B. Polée, J. F. Pruijt, G. Roeyen, J. A. Ropela, M. W. J. Stommel, J. de Vos-Geelen, J. J. de Vries, E. M. van der Waal, F. J. Wessels, J. W. Wilmink, H. C. van Santvoort, M. G. Besselink, I. Q. Molenaar, for the Dutch Pancreatic Cancer Group

**Affiliations:** 1 Departments of Surgery, Radiology and Medical Oncology, UMC Utrecht Cancer Center and St Antonius Hospital Nieuwegein: Regional Academic Cancer Center Utrecht, Utrecht University, Heidelberglaan 100, 3584 CX Utrecht, The Netherlands; 2 Departments of Surgery, Radiology and Medical Oncology, Cancer Center Amsterdam, Amsterdam UMC, University of Amsterdam, Amsterdam, The Netherlands; 3 Departments of Surgery and Medical Oncology, Jeroen Bosch Hospital, ‘s-Hertogenbosch, The Netherlands; 4 Departments of Surgery and Medical Oncology, Catharina Hospital, Eindhoven, The Netherlands; 5 Departments of Surgery and Medical Oncology GROW – School for Oncology and Developmental Biology, Maastricht UMC+, Maastricht, The Netherlands; 6 Departments of Surgery and Medical Oncology, OLVG, Amsterdam, The Netherlands; 7 Pancreatic Surgery Unit, Division of Surgery, CLINTEC, Karolinska Institute at Center for Digestive Diseases, Karolinska University Hospital, Stockholm, Sweden; 8 Departments of Surgery and Medical Oncology, UMC Groningen, Groningen, The Netherlands; 9 Departments of Surgery and Medical Oncology, Isala, Zwolle, The Netherlands; 10 Department of General and Digestive Surgery, Groeninge Hospital, Kortrijk, Belgium; 11 Departments of Surgery and Medical Oncology, Medical Spectrum Twente, Enschede, The Netherlands; 12 HBP Surgery and Transplant Department, Hospital Universitari Vall d’Hebron, Barcelona, Spain; 13 Department of Medical Oncology, Medical Center Leeuwarden, Leeuwarden, The Netherlands; 14 Department of Hepatobiliary, Endocrine and Transplantation Surgery, Antwerp University Hospital, Antwerp, Belgium; 15 Department of Medical Oncology, St Jansdal Hospital, Harderwijk, The Netherlands; 16 Department of Surgery, Radboud University Medical Center, Nijmegen, The Netherlands

**Keywords:** Locally advanced pancreatic cancer, Radiofrequency ablation, Chemotherapy, Overall survival

## Abstract

**Background:**

Approximately 80% of patients with locally advanced pancreatic cancer (LAPC) are treated with chemotherapy, of whom approximately 10% undergo a resection. Cohort studies investigating local tumor ablation with radiofrequency ablation (RFA) have reported a promising overall survival of 26–34 months when given in a multimodal setting. However, randomized controlled trials (RCTs) investigating the effect of RFA in combination with chemotherapy in patients with LAPC are lacking.

**Methods:**

The “Pancreatic Locally Advanced Unresectable Cancer Ablation” (PELICAN) trial is an international multicenter superiority RCT, initiated by the Dutch Pancreatic Cancer Group (DPCG). All patients with LAPC according to DPCG criteria, who start with FOLFIRINOX or (nab-paclitaxel/)gemcitabine, are screened for eligibility. Restaging is performed after completion of four cycles of FOLFIRINOX or two cycles of (nab-paclitaxel/)gemcitabine (i.e., 2 months of treatment), and the results are assessed within a nationwide online expert panel. Eligible patients with RECIST stable disease or objective response, in whom resection is not feasible, are randomized to RFA followed by chemotherapy or chemotherapy alone. In total, 228 patients will be included in 16 centers in The Netherlands and four other European centers. The primary endpoint is overall survival. Secondary endpoints include progression-free survival, RECIST response, CA 19.9 and CEA response, toxicity, quality of life, pain, costs, and immunomodulatory effects of RFA.

**Discussion:**

The PELICAN RCT aims to assess whether the combination of chemotherapy and RFA improves the overall survival when compared to chemotherapy alone, in patients with LAPC with no progression of disease following 2 months of systemic treatment.

**Trial registration:**

Dutch Trial RegistryNL4997. Registered on December 29, 2015. ClinicalTrials.govNCT03690323. Retrospectively registered on October 1, 2018

## Background

Pancreatic cancer is among the most deadliest of cancers with a worldwide incidence of approximately 460,000 new cases and 430,000 deaths in 2018 [1]. Approximately 80–90% of patients have no curative options due to metastatic disease or local tumor invasion into adjacent structures, i.e., locally advanced pancreatic cancer (LAPC) [2, 3]. Unfortunately, treatment options for patients with LAPC are limited. In patients with advanced pancreatic cancer, systemic treatment with gemcitabine monotherapy was found to improve quality of life compared to 5-FU and resulted in a median survival of 10–12 months in patients with LAPC [4–6]. FOLFIRINOX (a combination of 5-fluorouracil, oxaliplatin, irinotecan, and leucovorin) and nab-paclitaxel/gemcitabine showed a 4- and 2-month survival benefit, respectively, compared to gemcitabine monotherapy in patients with metastatic pancreatic cancer [7, 8]. Although no randomized controlled trials (RCTs) were performed, in patients with LAPC, both chemotherapy regimens have become generally accepted as the standard treatment [9]. Observational studies report an overall survival, according to intention-to-treat analyses, of 24 months for selected patients with LAPC after FOLFIRINOX and 19 months with nab-paclitaxel/gemcitabine [10, 11].

The first study on radiofrequency ablation (RFA) in patients with pancreatic cancer was published in 2000 [12]. RFA is a thermal-based local ablative therapy aiming for tumor destruction through the application of a high-frequency alternating current through one or more electrodes implanted into the tumor [13]. The principle of RFA for pancreatic cancer is essentially a form of tumor debulking rather than total tumor ablation, since several nearby vital structures are at risk. Overall complications and mortality were reported in 26% and 3%, respectively, after developing a method that leaves a peripheral rim of tumor as a safety margin to surrounding tissues [14]. Since then, several non-randomized studies have demonstrated RFA to be feasible and safe [15, 16]. When RFA was performed in a multimodal setting, combined with chemo(radio)therapy, a promising survival of 26–34 months was reported from single-center observational studies [17]. To objectively establish a survival benefit for RFA in LAPC in the current era of improved chemotherapy regimens, a RCT is needed.

## Aim

The “Pancreatic Locally Advanced Unresectable Cancer Ablation (PELICAN)” trial aims to compare median overall survival after a combination of chemotherapy with RFA versus chemotherapy alone, in patients with LAPC.

## Objectives and methods

The study objectives are to:

- Determine whether the combination of RFA and chemotherapy improves the overall survival for patients with LAPC, compared to chemotherapy alone
- Determine the effect of RFA combined with chemotherapy on pain, disease progression, tumor markers, and quality of life
- Evaluate the complications of RFA as well as the toxicity of chemotherapy and to estimate the costs of both treatment arms

### Study design

The PELICAN trial is an international multicenter parallel-group superiority RCT, initiated by the Dutch Pancreatic Cancer Group (DPCG).

### Study population

All patients with LAPC according to the National Comprehensive Cancer Network (NCCN) criteria (Table 1), without progression of disease who completed four cycles of FOLFIRINOX or two cycles of (nab-paclitaxel/)gemcitabine and are technically eligible for RFA, will be screened for study eligibility [18]. In addition, those patients with NCCN borderline resectable disease after chemotherapy, based on pre-operative imaging, who are found to be unresectable during explorative laparotomy due to local extension of disease, will be eligible for study inclusion.

**Table 1:** Definitions of the National Comprehensive Cancer Network (NCCN) and Dutch Pancreatic Cancer Group (DPCG) for locally advanced pancreatic cancer

	Arterial involvement	Venous involvement
NCCN criteria	SMA and celiac trunk involvement > 180°, aortic involvement	Unreconstructable PV/SMV occlusion
DPCG criteria	SMA, celiac trunk or hepatic artery involvement > 90°	PV/SMV involvement > 270° or occlusion

*NCCN* National Comprehensive Cancer Network, *DPCG* Dutch Pancreatic Cancer Group, *SMA* superior mesenteric artery, *PV* portal vein, *SMV* superior mesenteric vein

### Eligibility

Inclusion criteria are as follows:

1. Age ≥ 18 years
2. Histologically or cytologically confirmed or suspected pancreatic ductal adenocarcinoma
3. Locally unresectable tumor based on imaging according to NCCN criteria or unresectable during explorative laparotomy
4. Stable disease or partial response after four cycles of FOLFIRINOX or two cycles of (nab-paclitaxel/)gemcitabine (i.e., 2 months of treatment), according to the Response Evaluation Criteria in Solid Tumors v1.1 (RECIST) [19] and evaluated by the expert panel
5. Fit for surgery assessed by the treating surgeon and anesthesiologist
6. Fit for chemotherapy as assessed by the medical oncologist, plus the following:
  - Absolute neutrophil count ≥1.5 × 10^9^/L
  - Platelet count ≥100 × 10^9^/L
  - Renal function: creatinine clearance > 50 ml/min
  - AST/ALT ≤3× the upper limit of normal
7. RFA must be technically feasible (Additional file 1), assessed by an interventional radiologist from the expert panel
8. Written informed consent

Exclusion criteria are as follows:

1. World Health Organization (WHO) performance status ≥3
2. Distant metastases on abdominal or thoracic computed tomography (CT) scan
  - Lymph nodes are considered distant metastases depending on their location according to the International Study Group of Pancreatic Surgery, and only when pathologically proven [20].
3. Previous surgical resection, local ablative, radio- or chemotherapy for pancreatic cancer, other than the protocolled four cycles FOLFIRINOX or two cycles (nab-paclitaxel/)gemcitabine
4. A concomitant stenosis of > 50% of the hepatic artery and the portal or superior mesenteric vein
5. A second primary malignancy, except adequately treated non-melanoma skin cancer; in situ carcinoma of the cervix uteri; or other malignancies treated at least 5 years previously without signs of recurrence
6. Pregnancy

### Registration and randomization

**Fig. 1:**
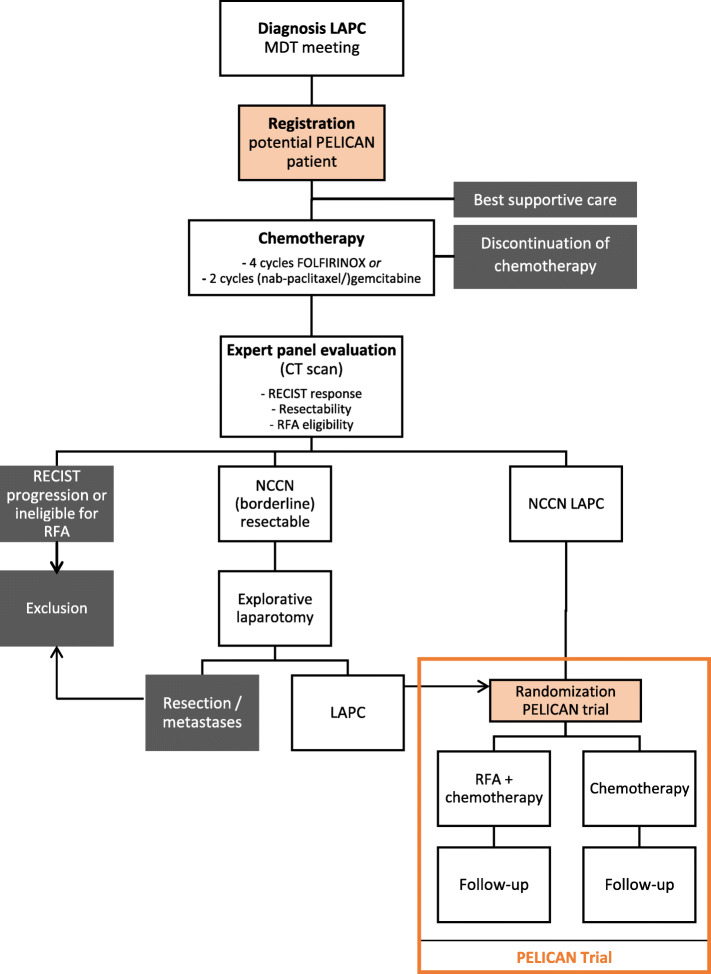
Trial flow diagram

Figure 1 shows the trial flow diagram. Patients will be identified for potential eligibility during the multidisciplinary team meeting at diagnosis. All patients with LAPC, based upon imaging, according to the DPCG criteria (Table 1) will be asked for informed consent for registration by a study coordinator, research nurse, or principal investigator. Patients will be treated in accordance with the standard of care and will either start chemotherapy or best supportive care, based on the advice of the multidisciplinary team meeting and shared decision-making between patient and a medical oncologist. In case of jaundice, patients will preferably receive a covered metal stent prior to induction therapy [21]. Patients who complete four cycles of FOLFIRINOX or two cycles of (nab-paclitaxel/)gemcitabine will be restaged with a CT scan of the chest and abdomen according to a standardized biphasic contrast-enhanced protocol. A nationwide expert panel consisting of abdominal radiologists, pancreatic surgeons, and interventional radiologists will review all restaging CT scans to evaluate the response to chemotherapy (RECIST v1.1), potential surgical resectability (NCCN criteria), and technical eligibility for RFA (Additional file 1) [18, 19]. Patients with progressive disease and those patients that are not technically eligible for RFA as determined by the expert panel are excluded. Patients who will be determined as borderline resectable at restaging, according to the NCCN guidelines, will undergo an explorative laparotomy with the intention of curative resection. If the tumor is found to be locally unresectable during surgery, the patient will be randomized intra-operatively for either RFA plus chemotherapy or chemotherapy alone. Prior to the explorative laparotomy, all patients must have provided written informed consent. The majority of patients, however, will be the group with NCCN unresectable, stable disease at restaging. The latter category of patients will be randomized at the outpatient clinic after obtaining written informed consent. Randomization will be performed centrally using a computer-generated randomization schedule randomization module (ALEA, Clinical Research Unit) in a 1:1 ratio between the following:

- Intervention-arm: RFA during laparotomy followed by a continuation of chemotherapy
- Control-arm: direct continuation of pre-randomization chemotherapy

Randomization will be stratified by institute and chemotherapy regimen.

### Intervention: radiofrequency ablation

Patients will be scheduled for surgery within 4 weeks after the restaging CT scan. All patients will receive antibiotic prophylaxis to prevent surgical site infections (cefazolin 2 g/metronidazole 500 mg) and will be administered a long-acting analog of somatostatin. The surgical procedure will be started with an exploratory laparoscopy to evaluate the presence of liver or peritoneal metastases. When there are no pathologically confirmed metastases, in the same session, a median laparotomy will be performed. The pancreas will be exposed by the Kocher maneuver. In case a tumor appears to be resectable during exploration and/or intra-operative ultrasound, conversion to a resection will be performed. When LAPC is confirmed intra-operatively, a cold wet gauze will be placed over the vena cava to prevent potential thermal damage. When a metal stent was placed during the pre-operative period, this is preferably removed first. Then, a RFA probe will be positioned by the interventional radiologist under direct ultrasound guidance, taking into account a prescribed safety zone to vital structures aiming for maximal tumor debulking rather than total tumor ablation (Additional file 1). A tumor biopsy will be taken intra-operatively from the center of the tumor, before and after RFA, as well as blood samples to measure the immunomodulatory factors. RFA will be carried out with the multipolar CelonLab® POWER System generator, Celon Aquaflow®, and bipolar Celon-ProSurge® (micro) applicators with exposure lengths of 9/15/20/30/40 mm (Olympus Surgical Technologies Europe, Teltow, Germany). A total of 15 kJ per probe will be delivered with a power setting of 1 W per millimeter probe length for probes 20–40 mm and 0.5 and 0.9 kJ with a power of 3 and 5 W for 9- and 15-mm probes, respectively [16, 22–24]. During RFA, the duodenum will be continuously perfused with cold saline through two nasogastric tubes to prevent thermal damage. One outflow tube will be placed directly post-pyloric, whereas the inflow tube will be placed in the duodenum near the ligament of Treitz to ensure a continuous duodenal flow with cold saline. A bowel clamp will be placed at the proximal jejunum to prevent cold saline to flow towards the ileum. RFA will be followed by a hepaticojejunostomy in all cases of a pancreatic head tumor. In case of expected duodenal obstruction, a gastric bypass (gastrojejunostomy) will be performed. An abdominal drain will be placed within the omental bursa, and the abdomen will be closed. After surgery, patients will be treated for 4 weeks with omeprazole 40 mg and thrombosis prophylaxis. Amylase will be measured from the drain fluid at day 1 and day 3, and a biphasic CT scan of the abdomen will be performed 7 days after the RFA procedure to visualize the RFA effect and to have a baseline scan before restarting chemotherapy. The additional chemotherapy schedule will be resumed as soon as patients are recovered from the RFA procedure.

### Control: chemotherapy alone

Patients will continue the chemotherapy treatment which was started after diagnosis, based on the advice of the multidisciplinary team meeting and shared decision-making between patient and a medical oncologist. In general, patients with a WHO performance status 0–1 and serum bilirubin levels ≤1.5 times the upper limit of the normal value will receive FOLFIRINOX or nab-paclitaxel/gemcitabine. If these criteria are not met, mostly nab-paclitaxel/gemcitabine or gemcitabine monotherapy will be administered. The objective is to administer a further 8 cycles of FOLFIRINOX after randomization or a further 4 cycles of (nab-paclitaxel/)gemcitabine. Details on chemotherapy administration are described in Additional file 2.

During chemotherapy, response evaluation with biphasic CT scans of the chest and abdomen will be performed after every four cycles of FOLFIRINOX or every two cycles of (nab-paclitaxel/)gemcitabine (i.e., 2 months).

### Study endpoints and definitions

The primary endpoint is overall survival by intention to treat, defined as the period of time between randomization and death by any cause. Patients alive at the last follow-up will be (right-)censored. Secondary endpoints are progression-free survival and radiologic tumor response according to RECIST v1.1, CA 19.9 and CEA response, toxicity according to the National Cancer Institute (NCI) Common Terminology Criteria (CTC) for adverse events v 4.0, quality of life (QLQ-C30, PAN-26), pain (visual analog scale), immunomodulatory effects (TNF-a, IL-8, IL-1-a, IL-1-b, MCP-1, IL-6, IL-33, DAMPs, and phenotyping), and costs [19, 25–27]. Progression-free survival is defined as the period of time between randomization and the date of local/regional progression, established on CT imaging, or occurrence of distant metastases or occurrence of a second pancreatic cancer or death [28]. Patients will be censored if a new anti-cancer therapy will be started prior to the documented progression or if two or more response assessments will be missed prior to a visit which documented the progression. In the RFA arm, postoperative complications are scored according to the International Study Group of Pancreatic Surgery [29–32].

### Data collection and follow-up

**Fig. 2:**
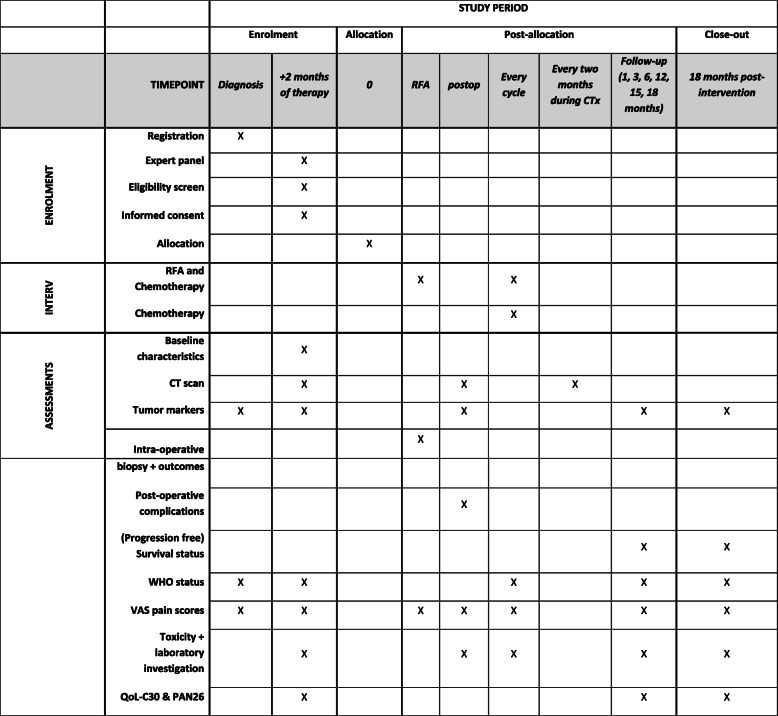
Schedule of enrolment, interventions, and assessments according to the SPIRIT guidelines

The selection of patients included in the trial will be made transparent by collecting reason for ineligibility for all registered patients with diagnosis LAPC that are excluded during the trial workup. After trial inclusion, baseline characteristics will be collected using standardized case record forms comprising age, sex, medical history, tumor markers, laboratory results, pre-randomization treatment, tumor characteristics (tumor size, location, vascular involvement), response to treatment, WHO performance status, body mass index, pain (visual analog scale), and quality of life. Treatment characteristics include chemotherapy dosage, modifications including reasons, toxicity, RFA procedural details (e.g., number of probes, distance to vital structures on intra-operative ultrasound, power settings, bypass surgery), and postoperative time to discharge and complications. After randomization, patients will be followed up at 1, 3, 6, 12, and 18 months after the start of the study treatment (i.e., date of RFA in group A and date of continuation of chemotherapy in group B). The follow-up consists of medical history including pain scores and WHO performance status, laboratory values (including tumor markers), and quality of life questionnaires. Furthermore, during chemotherapy, a biphasic CT scan of the chest and abdomen will be performed every 2 months for response evaluation. After completion of chemotherapy, CT scans will only be performed when indicated (i.e., complaints) (see Fig. 2 for a schedule of data collection and follow-up according to SPIRIT recommendations [33]). Due to the nature of the intervention, neither participants nor staff can be blinded to the allocation.

### Quality and safety

All participating centers that will perform RFA must have an available interventional radiologist or surgeon who routinely applies ultrasound-guided RFA procedures (e.g., for liver tumors) [34]. Furthermore, all participating centers had performed pancreatic surgery [35]. To ensure the quality of the implementation of the RFA pancreas, a RFA workshop was organized by the UMC Utrecht and Amsterdam UMC prior to the start of the study, during which specialists received a hands-on training in the execution of RFA procedures by proctors from Verona during two surgical procedures. Furthermore, in each participating hospital, at least the first two RFA procedures will be performed in the presence of an interventional radiologist of the trial’s expert panel. The exact frequency of the proctored procedures will be tailored based on the local expertise as assessed by the experienced proctor together with the participating center.

All (serious) adverse events ((S)AE) up to 28 days after the last protocol treatment will be recorded, except those directly related to the progression of the disease. SAEs will be reported to the principal investigator within 24 h, and within 15 days to the accredited medical ethical committee that approved the protocol. When a SAE results in death, it will be reported within 7 days after notification. (S)AEs will be reported through a web portal to the central committee on research involving human subjects (CCMO) and the accredited institutional review board.

In order to ensure the quality of the study, data collection and study monitoring will be performed by an independent research agency: the IKNL clinical research department. Pre-defined case report forms can be found at www.dpcg.nl/studie/pelican-2. The study monitor will have full access to the data to monitor the progress of the trial, capture and report data, and monitor the implementation in accordance with the protocol and Good Clinical Practice (GCP) standards. The monitoring plan includes verification of informed consent documents, checking in- and exclusion criteria for the first 10 patients per center and 25% afterwards, source data verification of 25% of included patients, regular on-site monitoring (twice a year per center, depending on patient enrollment), checking of adverse events in 10–25% of cases, and verification of essential documents within the investigator site file. Within each center, a local data manager is placed, responsible for including data within an electronic web-based database, query response, and communication with the central study monitor.

An independent data safety monitoring board (DSMB) consisting of at least a statistician or epidemiologist, surgeon, and gastroenterologist will monitor the safety of the trial subjects. Safety analyses will be held after each 20% of the sample size has completed the follow-up period. One formal interim analysis for efficacy will be performed after 85 events (i.e., death from any cause). The advice of the data safety monitoring board meeting will be shared with the steering committee and the ethical board of the trial.

### Sample size

Randomized controlled trials in patients with LAPC reported an average median survival of 10.4 months for patients receiving gemcitabine [5, 6, 36]. During the design of the study (2014), the literature on FOLFIRINOX and nab-paclitaxel/gemcitabine only include patients with metastatic disease and described a survival of 11.1 and 8.5 months, respectively [7, 8]. A 3-month survival difference was seen for patients treated with gemcitabine monotherapy when comparing LAPC with metastatic disease [5–8, 36]. This difference was extrapolated to LAPC patients treated with FOLFIRINOX and nab-paclitaxel resulting in an estimated survival of 14.1 and 11.5 months after FOLFIRINOX and nab-paclitaxel/gemcitabine, respectively. Taking into account that FOLFIRINOX was expected to be the most prescribed regimen, and taken into account an estimated time of 2–3 months between the start of chemotherapy and randomization, a survival of 10.2 months from randomization was estimated for the control group. Regarding the experimental study arm, a median survival benefit of at least 5.5 months with RFA + chemotherapy treatment was considered clinically worthwhile. Considering the time from randomization, this would translate into a median survival of 15.7 months, corresponding to a hazard ratio of 0.65. In order to have 80% power to detect a 35% reduction in the risk of death if RFA is added to chemotherapy, with a 1-sided 2.5% trial-wise type I error rate, a total of 169 events (death of any cause) need to be observed. Assuming a 2-year patient accrual period and a final analysis after another 18 months, a total of 228 patients need to be randomized in a 1:1 ratio, allowing an interim analysis after approximately half (1/2) of the total number of events.

### Statistical analysis

All randomized patients will be included in the analysis of overall survival and progression-free survival, according to the intention-to-treat principle. The final analysis on overall survival will be performed after having observed 169 events at about 42 months at a 2.45% 1-sided significance level, adjusted for the interim analysis. In addition, per-protocol analyses will be performed. Kaplan-Meier curves for proportions of event-free patients in each treatment arm will be calculated. The 95% confidence intervals for the median of time to event endpoints will be computed using the method of Brookmeyer and Crowley. In the primary analysis, the two treatment arms will be compared using the log-rank test stratified by the stratification factor except for center. The treatment effect and its 95% confidence interval will be estimated from the Cox regression model, stratified by the stratification factor except for center. In addition, the effect of the study center and other potential prognostic factors, such as the location of the tumor on the overall survival will be assessed using Cox regression. The Schoenfeld residual plots will be used to check the model assumption for the Cox regression.

Secondary outcomes will be examined using descriptive statistics, using the mean with standard deviation or median with interquartile range when appropriate for continuous data and number with percentage for categorical data. Comparison between the groups will be done with the chi-square tests and independent sample *t* tests when appropriate. Changes in the quality of life scores while on treatment versus baseline will be examined on specific time points to explore the treatment side effects on patients’ QoL and the longtime benefit of the study treatment. Baseline scores will be compared using a Wilcoxon rank sum test, and a pattern mixture model identifying dropout patients as a special category will be performed to evaluate the effect of missing data [37].

### Premature termination of the study

Based on the Lan-DeMets error spending function with O’Brian-Fleming type of boundaries, a significant benefit from RFA with chemotherapy is claimed if a *p* value of less than 0.00153 in favor of RFA with chemotherapy will be observed at the interim analysis. Furthermore, an independent data safety monitoring board will analyze the safety and may advise the trial steering committee to adjust or stop the study prematurely in case of safety concerns, taking the study outcome into account.

### Modification of the protocol

Any modifications to the protocol which may impact the conduct of the study, potential benefit, or safety of the patient, including changes of study objectives, study design, patient population, sample sizes, study procedures, or significant administrative aspects, will require a formal amendment to the protocol. Additional file 3 includes all amendments until July 2020 that were all approved by the Ethics Committee prior to implementation.

### Dissemination policy

The trial results will be submitted for presentation at (inter)national conferences (i.e., International Hepato-Pancreato-Biliary Association (IHPBA), the Americas HPBA (AHPBA), European-African HPBA (E-AHPBA), European Pancreatic Club, Pancreas Club Annual Meeting) and publication in a peer-reviewed journal, regardless of the outcome. When positive trial results will be established, other centers that perform pancreatic surgery will be proctored by interventional radiologists from the PELICAN expert panel. Extensive experience with proctoring in national and international centers within the trial has already been gained. Moreover, RFA of the pancreas should only be implemented in centers that routinely apply ultrasound-guided RFA procedures (e.g., for liver tumors).

Co-authorship will be based on the international ICMJE guidelines, with at least one co-authorship per site (internally determined) and more depending on the inclusion rate. Furthermore, all the members of the protocol writing committee will be awarded with an authorship after revising the work critically, since they substantially contributed to the conception and design of this study.

## Discussion

The PELICAN trial is an international multicenter randomized controlled superiority trial designed to assess whether in patients with LAPC, RFA in combination with chemotherapy improves the overall survival as compared with chemotherapy alone.

In preparation for the trial, surgeons and interventional radiologists of the principal study sites were trained by the expert group in Verona, including a visit in Italy and on-site proctoring in The Netherlands. Afterwards, the study group performed two experimental studies and a phase II clinical safety study to assess the safety and the effect of the RFA settings [16, 23, 24]. As described in the “Objectives and methods” section, an extensive proctoring plan was designed, to further secure the quality and safety of the study procedure. With these results and measures, it was decided together with the Dutch Pancreatic Cancer Group (DPCG) that a randomized controlled phase III trial was justified and safe and the PELICAN trial was designed.

During the design phase of the PELICAN trial, the timing of restaging within the expert panel and consideration for trial inclusion as well as explorative laparotomy was a matter of debate. In earlier days, when standard treatment for patients with LAPC was gemcitabine monotherapy, most studies performed RFA as upfront therapy [14, 16]. However, in the current era of FOLFIRINOX, a more pronounced improvement of overall survival and also the possibility of a resection after chemotherapy are described [38, 39]. In addition, studies that investigated RFA as part of a multimodal treatment strategy showed improved overall survival up to 34 months [40]. Therefore, it was decided to investigate the effect of RFA only after a period with one of the standard chemotherapy regimens. Based on consensus meetings and a survey among the participating medical oncologists, it was decided to include patients after the first response evaluation after approximately 2 months of treatment. It was expected that dropout due to toxicity was minimal at this moment. This was also consistent with the largest published cohort at that moment where 75% of consolidation therapy was started after the first tumor evaluation [41]. Current studies focusing on resection after induction chemotherapy mostly advise a period of 4 to 6 months before proceeding to a surgical explorative laparotomy, which is longer than defined in the PELICAN trial protocol. This might suggest that patients included in the trial are withheld a possible surgical resection. However, we do not yet know the ideal timing of an explorative laparotomy since these advices are all based on expert opinions. Also, after the inclusion in the trial, patients will receive response evaluations with a CT scan every 2 months and can proceed to a surgical exploration even after randomization within the study [9]. This might introduce bias when an imbalance between resections will arise between treatment arms. This can also result from the explorative laparotomy in advance of the RFA procedure, in which patients might undergo a resection. In order to minimize this bias, patients with potential resectable disease are randomized intra-operatively, after unresectability has been established. Moreover, a per-protocol analysis and Cox regression analysis will be done to investigate and eliminate this potential effect on overall survival.

During the design of the study, it was discussed whether a staging laparoscopy for all patients prior to randomization was needed, since occult metastases are present in up to 19% of patients with LAPC and these patients are not eligible for radiofrequency ablation [42]. However, since patients are included after induction chemotherapy, it is uncertain whether these metastases will be detectable. Moreover, within the control arm, it would have no consequences when occult metastases will be found. Therefore, it was decided unethical to perform an invasive procedure without consequences in at least 50% of patients (control arm). It can be assumed that due to randomization, patients with occult metastases are equally distributed between the groups. Since the results will be analyzed according to the intention-to-treat principle, this bias will influence both groups equally. In recently published studies, the median overall survival of patients with LAPC treated with FOLFIRINOX seems longer than 14.1 months as assumed during the sample size calculation. A recent meta-analysis reported a median overall survival of 24 months for patients with LAPC treated with (modified) FOLFIRINOX [11]. Although it was taken into account that the population included within the trial will be a favorable selection of patients, this suggests an underestimated survival within the control arm. If true, this results in an underpowered study with the current sample size. However, the studies included within the meta-analysis are mostly single-center studies from experienced centers and also included patients receiving FOLFIRINOX as a multimodal treatment strategy in combination with a resection or (chemo)radiotherapy [38, 43, 44]. Different definitions for LAPC are used, and the external validity of these results is uncertain. A recent observational study including 680 consecutive patients with borderline resectable and LAPC showed a median overall survival of 13 months for all patients after an intention-to-treat analysis [45]. Recent multicenter data from The Netherlands showed a median overall survival of 14 months for patients with LAPC treated with FOLFIRINOX [46]. These studies likely better reflect “real-world” data, and the assumption of 14.1 months can be preserved with these data.

Obviously, due to the nature of the study with a non-surgical control arm and a surgical intervention, it is impossible to blind patients and treating physicians. Therefore, performance and ascertainment bias might be introduced for subjective secondary outcomes like quality of life and pain scores, and these results must thus be interpreted with care. Furthermore, a practical issue that will be challenging is the multicenter nature of the study combined with the pre-randomization registration in which many potential patients need to be followed in order to include only those that are eligible. Other pending randomized controlled trials that investigate ablative treatment strategies in patients with LAPC are the CROSSFIRE trial (ClinicalTrials.gov NCT02791503), DIRECT trial (ClinicalTrials.gov NCT03899649), and the PANC0015 trial (ClinicalTrials.gov NCT01926197). The latter is the only other registered study that compares an ablative therapy directly with chemotherapy in a randomized setting and inclusion currently stopped due to low accrual. This affirms the difficulty of performing a randomized controlled trial within this specific patient population and emphasizes the importance to perform this trial with a large multicenter collaboration like the Dutch Pancreatic Cancer Group [47]. Within this multidisciplinary organization, there is a lot of experience with multicenter studies, and together with a data management grant from the Dutch Cancer Society, we are confident that we will have enough resources to manage the trial and complete it successfully.

### Trial status

The first patient was randomized on April 7, 2015. At the time of protocol submission (July 2020), protocol version 10.2 (March 6, 2018) was effective and 16 centers in The Netherlands and 3 centers in Belgium and Spain were actively recruiting patients for the trial. One hundred forty-nine of 228 patients (65%) have been randomized (see www.dpcg.nl/studie/pelican-2 for the up-to-date information on participating centers and the number of included patients). Inclusion is behind schedule which is partly related to a higher than expected proportion of patients undergoing surgical resection with curative intent and more patients than expected being ineligible for RFA. This was discussed within the Medical Ethical Committee and Data Safety Monitoring Board and Grant provider (Dutch Cancer Society) in 2017 and 2018. Since the PELICAN trial is the only ongoing randomized controlled trial worldwide on this specific topic, all acknowledged the importance of the trial. To improve patient accrual, 3 more centers in Europe were opened for inclusion. It is estimated that recruitment will be completed in December 2021.

## Supplementary Information

**Additional file 1.** Eligibility criteria RFA.

**Additional file 2.** Chemotherapy administration.

**Additional file3.** Protocol amendments and approval by METC.

**Additional file 4.** SPIRIT checklist.

**Additional file 5.** Translation grant.

**Additional file 6.** Translation METC document.

## Data Availability

Not applicable, no datasets are included in this study protocol.

## Abbreviations

LAPC: Locally advanced pancreatic cancer
RFA: Radiofrequency ablation
RCT: Randomized controlled trial
DPCG: Dutch Pancreatic Cancer Group
RECIST: Response Evaluation Criteria In Solid Tumors
NCCN: National Comprehensive Cancer Network
WHO: World Health Organization
CT: Computed tomography
NCI CTC: National Cancer Institute Common Terminology Criteria
SAE: Serious adverse event
GCP: Good Clinical Practice
DSMB: Data Safety Monitoring Board
