# Supplementary material for: Radiofrequency ablation and chemotherapy versus chemotherapy alone for locally advanced pancreatic cancer (PELICAN): study protocol for a randomized controlled trial
Source: Trials. 2021 Apr 29;22:313. doi: 10.1186/s13063-021-05248-y (PMC8082784; doi:10.1186/s13063-021-05248-y)
Supplement: Supplementary file 1 — Additional file 1. Eligibility criteria RFA. [file 13063_2021_5248_MOESM1_ESM.docx]

**Additional file 1. Eligibility criteria RFA**

An ablation can be performed, when the edge of the estimated ablation zone has a safety distance of at least 5 mm to the vital structures (portal vein, superior mesenteric vein, superior mesenteric artery, common hepatic artery, celiac trunk, caval vein, duodenum). The size of the ablation zone is dependent on the electrode and ellipse shaped with a diameter of:

- T9 electrode – 6-8mm
- T15 electrode – 8-10mm
- T20-T40 electrodes – 20mm

Patients with a stenosis of both the portal vein/ superior mesenteric vein and the hepatic artery of > 50% are exluded based on the study teams experience of a case with acute thrombosis of the portal vein and liver failure as a consequence of a compromised hepatic artery.

The expert panel will assess whether patients meet these criteria based on biphasic CT-abdomen after induction chemotherapy. These criteria are based on the principle that the intention of the RFA of pancreatic tumors is essentially a form of tumor debulking rather than total tumor ablation. Besides, recent analysis of previous performed studies did not show a correlation between the size of the necrotic area and the prognosis of the patient.
