# Supplementary material for: Radiofrequency ablation and chemotherapy versus chemotherapy alone for locally advanced pancreatic cancer (PELICAN): study protocol for a randomized controlled trial
Source: Trials. 2021 Apr 29;22:313. doi: 10.1186/s13063-021-05248-y (PMC8082784; doi:10.1186/s13063-021-05248-y)
Supplement: Supplementary file 2 — Additional file 2. Chemotherapy administration. [file 13063_2021_5248_MOESM2_ESM.docx]

**Additional file 2. Chemotherapy administration**

FOLFIRINOX consists of oxaliplatin at a dose of 85 mg/m2, given as a 2-hour intravenous infusion, immediately followed by leucovorin at a dose of 400 mg/m2, given as a 2-hour intravenous infusion, with the addition, after 30 minutes, of irinotecan at a dose of 180 mg/m2, given as a 90-minute intravenous infusion. This treatment will immediately be followed by fluorouracil at a dose of 400 mg/m2, administered by intravenous bolus, followed by a continuous infusion of 2400 mg/m2 over a 46-hour period every two weeks. In total, patients will receive 8 cycles of FOLFIRINOX after randomization.

The regimen of nab-paclitaxel/gemcitabine consists of a 30-to-40-minute intravenous infusion of nab-paclitaxel at a dose of 125 mg/m2, followed by a 30-minute infusion of gemcitabine at a dose of 1000 mg/m2, on days 1, 8 and 15 every four weeks. After randomization patients will receive four cycles.

The gemcitabine cycle consists of a 30-minute intravenous infusion of gemcitabine at a dose of 1000 mg/m2 on day 1, 8 and 15 followed by one week of rest. Patients will be treated with four cycles after randomization.

All patients receive prophylactic anti-emetics.

**Modifications in the FOLFIRINOX regimen in case of deviations in neutrophils, platelets, renal and liver function**

|  | **Irinotecan** | | **Oxaliplatin** | **5FU** |
| --- | --- | --- | --- | --- |
| ***Neutrophils*** | | | | |
| **1^st^ x low neutrophils, febrile neutropenia or < 0.5 more than 7 days** | Reduce to  150 mg/m^2^ | | Full dose | Omit bolus |
| **2nd x abovementioned** | Maintain  150 mg/m^2^ | | Reduce to 60 mg/m^2^ | Omit bolus |
| **3^rd^ x abovementioned** | Stop treatment | | | |
| ***Platelets*** | | | | |
| **1^st^ x platelets < 75 *10^9^/L** | Full dose | | Reduce to 60 mg/m^2^ | Reduce bolus and continuous infusion to 75% |
| **2^nd^ x platelets < 75 *10^9^/L** | Reduce to 150 mg/m^2^ | | Maintain  60 mg/m^2^ | Reduce bolus and continuous infusion to 75% |
| **3^rd^ x platelets < 75 *10^9^/L** | Stop treatment | | | |
| ***Renal function*** | | | | |
| **Creat clearance**  **≥30 – <50 ml/min** | 75% | | 100% | Reduce bolus and continuous infusion to 75% |
| **Creatinine clearance < 30 ml/min** | 50% | | No oxaliplatin | 50% |
| ***Liver function*** | | | | |
| **bilirubine 1.5 – 3 x ULN, transaminasen> 5 x ULN** | | 50% | 100% | 100% |
| **Bilirubine> 3 x ULN** | | Non irinotecan | 50% | 50% |

**Stepwise dose reduction for nab-paclitaxel/gemcitabine**

| **Dose level** | **Nab-paclitaxel (mg/m^2^)** | **Gemcitabine (mg/m^2^)** | |
| --- | --- | --- | --- |
| **Full dose** | 125 | | 1000 |
| **1^st^ step dose reduction** | 100 | | 800 |
| **2^nd^ step dose reduction** | 75 | | 600 |
| **When further dose reduction is necessary** | Stop treatment | Stop treatment | |

**Dose reductions for nab-paclitaxel/gemcitabine in case of neutropenia or thrombocytopenia.**

| **Day of each cycle** | **Nab-paclitaxel** | **Gemcitabine** |
| --- | --- | --- |
| ***Day 1*** | |  |
| **ANC < 1.5x10^9^ / L OR platelets < 100x10^9^/L** | Delay by 1 week intervals until recovery | |
| ***Day 8*** |  |  |
| **500 ≤ ANC < 1000 OR 50.000 ≤ platelets < 75.000** | Reduce dose with 1 step | |
| **ANC < 500 OR platelets < 50.000** | Hold dose | |
| ***Day 15: If day 8: full dose was given*** | |  |
| **500 ≤ ANC < 1000 OR 50.000 ≤ platelets < 75.000** | Add WBC growth factors to treatment OR decrease dose with 1 step | |
| **ANC < 500 OR platelets < 50,000** | Hold dose | Hold dose |
| ***Day 15: If day 8: dose reduction*** | |  |
| **ANC ≥ 1000 AND platelets ≥ 75,000** | Return to dose given at day 1 + WBC growth factors OR same dose as day 8 | |
| **500 ≤ ANC < 1000 OR 50.000 ≤ platelets < 75.000** | Same dose as day 8 + WBC growth factors OR reduce dose with 1 step compared to day 8 | |
| **ANC < 500 or platelets < 50,000** | Hold dose | |
| ***Day 15: if day 8: dose was hold*** | | |
| **ANC ≥ 1000 and platelets ≥ 75,000** | Return to dose given at day 1 + WBC growth factors OR reduce with 1 step compared to day 1 | |
| **500 ≤ ANC < 1000 OR 50.000 ≤ platelets < 75.000** | Reduce dose with 1 step + WBC growth factors OR Reduce dose with two steps compared to day 1 | |
| **ANC < 500 or platelets < 50,000** | Hold dose | |

**Dose reductions for nab-paclitaxel/gemcitabine in case of other forms of toxicities**

| **Complication** | **Nab-paclitaxel** | **Gemcitabine** | |
| --- | --- | --- | --- |
| **Grade 3 or 4 peripheral neuropathy** | Hold dose until recovery to at least ≤ grade 1; resume dose reduced with one step. | | Treat with the same dose as before |
| **Grade 2 or 3 cutaneous toxicity** | Reduce dose with one step; stop treatment when toxicity persists | | |
| **Gastro-intestinal toxicity: grade 3 mucositis or diarrhea** | Hold dose until recovery to ≤ grade 1; resume dose reduced with one step. | | |

**Dose modifications of gemcitabine**

| **Absolute Neutrophil Count (10^9^/L)** |  | **Platelets (10^9^/L)** | **Gemcitabine dose (%)** |
| --- | --- | --- | --- |
| > 1.5 | AND | > 75 | 100 |
| ≥ 1.0 - < 1.5 | AND | > 50 - <75 | 75 |
| < 1.0 | OR | < 50 | Postponed |
