# Supplementary material for: Radiofrequency ablation and chemotherapy versus chemotherapy alone for locally advanced pancreatic cancer (PELICAN): study protocol for a randomized controlled trial
Source: Trials. 2021 Apr 29;22:313. doi: 10.1186/s13063-021-05248-y (PMC8082784; doi:10.1186/s13063-021-05248-y)
Supplement: Supplementary file 3 — Additional file3. Protocol amendments and approval by METC. [file 13063_2021_5248_MOESM3_ESM.docx]

**Additional file 3. Protocol amendments and approval by METC**

| **Date submitted** | **Date of approval** | **New documents** | **Summary** |
| --- | --- | --- | --- |
| 06-10-2014 | - | Protocol v1 | Never used, changed to v2 before approval |
| 19-12-2014 | 24-12-2014 | Protocol v2  PIF/IC v2 | Clarification patient information |
| 09-03-2015 | 20-03-2015 | Protocol v3 | Change protocol writing committee; Investigators; Add immunomodulation as endpoint;  Change in- exclusion criteria; criteria for chemotherapy changed; Qol in appendix |
| 13-04-2015 | - | Protocol v4 | Investigators UMCU, Radboud, RdGG; Change of in- and exclusion criteria; change aim to ablate >50% tumor **into** strive to create widest possible ablation.  not used changed to v6 before approval |
| 20-04-2015 | - | Protocol v5 | FU from start of study treatment instead of randomization. Add stenosis of both portal vein/SMV and hepatic artery as exclusion to appendix 2  Protocol not used changed to v6 before approval |
| 05-06-2015 | 18-06-2015 | Protocol v6 | Added study endpoint: time from randomization to start treatment. |
| 31-08-2015 | 11-11-2015 | Protocol v7  PIF&IC v5  IC FU v1 | Change study coordinator/investigators; criteria for registration; added side study Expect; changed exclusion criteria (portal vein thrombus, second malignancy); FU when patients go to non-PELICAN center for chemotherapy.  Added Expect, specify that chemotherapy needs to be given in PELICAN center.  Consent to come to outpatient clinics at FU moments. |
| 21-12-2015 | 12-01-2016 | Protocol v8 | Added nab-paclitaxel with change of sample size. Added UMCU to Expect side study. Specified when lymph nodes are considered as metastastic. Adjusted flow duodenal cooling at RFA procedure. |
| 18-04-2016 | 17-06-2016 | Protocol 9 | Clarification difference between locoregional lymph node metastases vs distance lymph node metastases.  Description Celon ProSurge micro Applicators.  Clarification criteria for nab-paclitaxel and gemcitabine. |
| 17-01-2018 | 26-03-2018 | Protocol 10 | Adjusting title due tuo international centers. Further clarification of dose reductions due to toxicity of FOLFIRINOX. |
|  |  |  |  |
