# Supplementary material for: Radiofrequency ablation and chemotherapy versus chemotherapy alone for locally advanced pancreatic cancer (PELICAN): study protocol for a randomized controlled trial
Source: Trials. 2021 Apr 29;22:313. doi: 10.1186/s13063-021-05248-y (PMC8082784; doi:10.1186/s13063-021-05248-y)
Supplement: Supplementary file 4 — Additional file 4. SPIRIT checklist. [file 13063_2021_5248_MOESM4_ESM.docx]

**Additional file 3. SPIRIT**
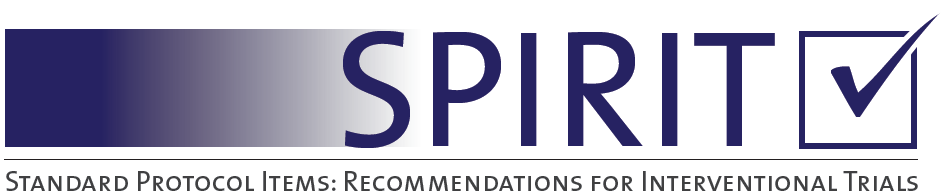
**checklist**

SPIRIT 2013 Checklist: Recommended items to address in a clinical trial protocol and related documents*

| Section/item | ItemNo | | Description |  | |
| --- | --- | --- | --- | --- | --- |
| **Administrative information** | | | | |  |
| Title | 1 | Descriptive title identifying the study design, population, interventions, and, if applicable, trial acronym | | | Yes, see title p1 |
| Trial registration | 2a | Trial identifier and registry name. If not yet registered, name of intended registry | | | Yes, see abstract, p2 |
|  | 2b | All items from the World Health Organization Trial Registration Data Set | | | Yes, see trialregister.nl or  clinicaltrials.gov, p2 |
| Protocol version | 3 | Date and version identifier | | | Yes, 10.2 (6 march 2018) see manuscript ‘trial status’, p17 |
| Funding | 4 | Sources and types of financial, material, and other support | | | Yes, see Funding, in  declaration section, p20 |
| Roles and responsibilities | 5a | Names, affiliations, and roles of protocol contributors | | | Yes, see title page p1 and authors contributions p20, in declaration section |
|  | 5b | Name and contact information for the trial sponsor | | | Yes, see ethics approval in declaration section, p18 |
|  | 5c | Role of study sponsor and funders, if any, in study design; collection, management, analysis, and interpretation of data; writing of the report; and the decision to submit the report for publication, including whether they will have ultimate authority over any of these activities | | | There was no such role,  See funding, in declaration  Section, p20 |
|  | 5d | Composition, roles, and responsibilities of the coordinating centre, steering committee, endpoint adjudication committee, data management team, and other individuals or groups overseeing the trial, if applicable (see Item 21a for data monitoring committee) | | | Yes, every center had a local  PI responsible for local pro-  cedures and safety monito-  ring. There was an  independent data monitor. See quality and safety section  p12 |
| Introduction |  |  | | |  |
| Background and rationale | 6a | Description of research question and justification for undertaking the trial, including summary of relevant studies (published and unpublished) examining benefits and harms for each intervention | | | Yes, see background, p4-5 |
|  | 6b | Explanation for choice of comparators | | | Intervention vs. standard of  Care, page 4-5 |
| Objectives | 7 | Specific objectives or hypotheses | | | Yes, see introduction, p5 |
| Trial design | 8 | Description of trial design including type of trial (eg, parallel group, crossover, factorial, single group), allocation ratio, and framework (eg, superiority, equivalence, noninferiority, exploratory) | | | Yes, see p5 |
| Methods: Participants, interventions, and outcomes | | | | |  |
| Study setting | 9 | Description of study settings (eg, community clinic, academic hospital) and list of countries where data will be collected. Reference to where list of study sites can be obtained | | | See registration and  Randomization. p17 |
| Eligibility criteria | 10 | Inclusion and exclusion criteria for participants. If applicable, eligibility criteria for study centres and individuals who will perform the interventions (eg, surgeons, psychotherapists) | | | Yes, see Eligibility in methods  Section, p6-7 |
| Interventions | 11a | Interventions for each group with sufficient detail to allow replication, including how and when they will be administered | | | Yes, see intervention and  Control in methods section  p8-9 |
|  | 11b | Criteria for discontinuing or modifying allocated interventions for a given trial participant (eg, drug dose change in response to harms, participant request, or improving/worsening disease) | | | Yes, see intervention p8 and  Additional file 2. |
|  | 11c | Strategies to improve adherence to intervention protocols, and any procedures for monitoring adherence (eg, drug tablet return, laboratory tests) | | | Mainly through telephone  Contact between study  Coordinator and physicians  and patients, p17 |
|  | 11d | Relevant concomitant care and interventions that are permitted or prohibited during the trial | | | Yes, see censoring reasons  p10 and eligibility criteria p6 |
| Outcomes | 12 | Primary, secondary, and other outcomes, including the specific measurement variable (eg, systolic blood pressure), analysis metric (eg, change from baseline, final value, time to event), method of aggregation (eg, median, proportion), and time point for each outcome. Explanation of the clinical relevance of chosen efficacy and harm outcomes is strongly recommended | | | Yes, see study endpoints  And definitions, p10 |
| Participant timeline | 13 | Time schedule of enrolment, interventions (including any run-ins and washouts), assessments, and visits for participants. A schematic diagram is highly recommended (see Figure) | | | Yes, see SPIRIT figure.  Figure 2, p28 |
| Sample size | 14 | Estimated number of participants needed to achieve study objectives and how it was determined, including clinical and statistical assumptions supporting any sample size calculations | | | Yes, see sample size in  methods section, p12 |
| Recruitment | 15 | Strategies for achieving adequate participant enrolment to reach target sample size | | | Yes, mainly through intensive  follow-up by study coordinator  p17 |
| **Methods: Assignment of interventions (for controlled trials)** | | | | |  |
| Allocation: |  |  | | |  |
| Sequence generation | 16a | Method of generating the allocation sequence (eg, computer-generated random numbers), and list of any factors for stratification. To reduce predictability of a random sequence, details of any planned restriction (eg, blocking) should be provided in a separate document that is unavailable to those who enrol participants or assign interventions | | | Yes, computer generated,  Online module by study  Coordinator or IKNL, p7 |
| Allocation concealment mechanism | 16b | Mechanism of implementing the allocation sequence (eg, central telephone; sequentially numbered, opaque, sealed envelopes), describing any steps to conceal the sequence until interventions are assigned | | | Yes, online module, p7 |
| Implementation | 16c | Who will generate the allocation sequence, who will enrol participants, and who will assign participants to interventions | | | Allocation sequence is  Generated by an independent  person not involved in  Assigning interventions or  Enrolling patients, p7 |
| Blinding (masking) | 17a | Who will be blinded after assignment to interventions (eg, trial participants, care providers, outcome assessors, data analysts), and how | | | NA, no blinding |
|  | 17b | If blinded, circumstances under which unblinding is permissible, and procedure for revealing a participant’s allocated intervention during the trial | | | NA, no blinding |
| **Methods: Data collection, management, and analysis** | | | | |  |
| Data collection methods | 18a | Plans for assessment and collection of outcome, baseline, and other trial data, including any related processes to promote data quality (eg, duplicate measurements, training of assessors) and a description of study instruments (eg, questionnaires, laboratory tests) along with their reliability and validity, if known. Reference to where data collection forms can be found, if not in the protocol | | | Yes, data management grant.  Data collection is done by an  Independent research agency: IKNL clinical research dpt. Predefined case record forms.  See p12 |
|  | 18b | Plans to promote participant retention and complete follow-up, including list of any outcome data to be collected for participants who discontinue or deviate from intervention protocols | | | Case record forms will be  used, also for those deviating  from intervention. p12 |
| Data management | 19 | Plans for data entry, coding, security, and storage, including any related processes to promote data quality (eg, double data entry; range checks for data values). Reference to where details of data management procedures can be found, if not in the protocol | | | Yes, see protocol, additional  File  (not in manuscript) |
| Statistical methods | 20a | Statistical methods for analysing primary and secondary outcomes. Reference to where other details of the statistical analysis plan can be found, if not in the protocol | | | Yes, see protocol and  Manuscript: Statistical  Analysis, p13 |
|  | 20b | Methods for any additional analyses (eg, subgroup and adjusted analyses) | | | see p13 |
|  | 20c | Definition of analysis population relating to protocol non-adherence (eg, as randomised analysis), and any statistical methods to handle missing data (eg, multiple imputation) | | | Yes, see protocol. Intention  to treat ánd per protocol  analyses, p13 |
| **Methods: Monitoring** | | | | |  |
| Data monitoring | 21a | Composition of data monitoring committee (DMC); summary of its role and reporting structure; statement of whether it is independent from the sponsor and competing interests; and reference to where further details about its charter can be found, if not in the protocol. Alternatively, an explanation of why a DMC is not needed | | | Yes, data monitoring  By IKNL clinical research  Department (data monitoring  Grant), p12 |
|  | 21b | Description of any interim analyses and stopping guidelines, including who will have access to these interim results and make the final decision to terminate the trial | | | Yes, see manuscript and  Protocol. p12 |
| Harms | 22 | Plans for collecting, assessing, reporting, and managing solicited and spontaneously reported adverse events and other unintended effects of trial interventions or trial conduct | | | Yes, see Safety section and  Data monitoring safety board  p11-12 |
| Auditing | 23 | Frequency and procedures for auditing trial conduct, if any, and whether the process will be independent from investigators and the sponsor | | | Yes every 20% DSMB will  Audit trial and safety. p12 |
| Ethics and dissemination | | | | |  |
| Research ethics approval | 24 | Plans for seeking research ethics committee/institutional review board (REC/IRB) approval | | | Yes, already approved, p18 |
| Protocol amendments | 25 | Plans for communicating important protocol modifications (eg, changes to eligibility criteria, outcomes, analyses) to relevant parties (eg, investigators, REC/IRBs, trial participants, trial registries, journals, regulators) | | | Yes, by e-mail and via  Website, NA for manuscript |
| Consent or assent | 26a | Who will obtain informed consent or assent from potential trial participants or authorised surrogates, and how (see Item 32) | | | Yes, at outpatient clinics, after  Identification at MDT. By  Research nurse or  Trial coordinator. P7 |
|  | 26b | Additional consent provisions for collection and use of participant data and biological specimens in ancillary studies, if applicable | | | Yes, integrated within the  Informed consent form. P7 |
| Confidentiality | 27 | How personal information about potential and enrolled participants will be collected, shared, and maintained in order to protect confidentiality before, during, and after the trial | | | Yes, see protocol +  Patient information |
| Declaration of interests | 28 | Financial and other competing interests for principal investigators for the overall trial and each study site | | | Yes, see manuscript  Declaration section, p18 |
| Access to data | 29 | Statement of who will have access to the final trial dataset, and disclosure of contractual agreements that limit such access for investigators | | | Yes, see protocol. |
| Ancillary and post-trial care | 30 | Provisions, if any, for ancillary and post-trial care, and for compensation to those who suffer harm from trial participation | | | Yes, insurance, included in  Pt information, p18 |
| Dissemination policy | 31a | Plans for investigators and sponsor to communicate trial results to participants, healthcare professionals, the public, and other relevant groups (eg, via publication, reporting in results databases, or other data sharing arrangements), including any publication restrictions | | | Yes, newsletters for trial  participants and family  will be made. Meetings for  trial professionals will be  organized. p14 |
|  | 31b | Authorship eligibility guidelines and any intended use of professional writers | | | Yes, see protocol. p14 |
|  | 31c | Plans, if any, for granting public access to the full protocol, participant-level dataset, and statistical code | | | NA; No plans are made to  Grant public access to the  dataset. |
| Appendices |  |  | | |  |
| Informed consent materials | 32 | Model consent form and other related documentation given to participants and authorised surrogates | | | Yes |
| Biological specimens | 33 | Plans for collection, laboratory evaluation, and storage of biological specimens for genetic or molecular analysis in the current trial and for future use in ancillary studies, if applicable | | | Yes, within biobank. |

*It is strongly recommended that this checklist be read in conjunction with the SPIRIT 2013 Explanation & Elaboration for important clarification on the items. Amendments to the protocol should be tracked and dated. The SPIRIT checklist is copyrighted by the SPIRIT Group under the Creative Commons “[Attribution-NonCommercial-NoDerivs 3.0 Unported](http://www.creativecommons.org/licenses/by-nc-nd/3.0/)” license.
