# Supplementary material for: Radiofrequency ablation and chemotherapy versus chemotherapy alone for locally advanced pancreatic cancer (PELICAN): study protocol for a randomized controlled trial
Source: Trials. 2021 Apr 29;22:313. doi: 10.1186/s13063-021-05248-y (PMC8082784; doi:10.1186/s13063-021-05248-y)
Supplement: Supplementary file 5 — Additional file 5. Translation grant. [file 13063_2021_5248_MOESM5_ESM.pdf]

University Medical Center Utrecht
t.a.v. dr. I.Q. Molenaar
Department of Surgery
Heidelberglaan 100
3508 GA Utrecht

Date 22th of December 2014
Reference TDM-H1/7444

Dear dr. Molenaar

Hereby I send you the advice of the scientific board of the Dutch Cancer Society concerning your clinical trial:

UU 2014-7444, titled: PELICAN trial, Pancreatic Locally advanced Irresectable Cancer Ablation in the Netherlands.

Information on the review process was also given within this document.

I am pleased to tell you that based upon the advice of the scientific board, the Dutch Cancer Society decided to assign a grant in order to execute your clinical trial. I ask you to read the advice and the grant conditions thoroughly.

Extent of the grant:
The advice of the scientific board concerning the amount of the grant can be found in the supplementary advice. The total amount will be 296.588 euro for data management and another 131.444 euro supplementary.

Different from previous years, you do not receive a 'grant acceptation statement'. Instead, within the grant conditions is written that by accepting the grant, the principal investigator(s) and research institute agree with the eligibility conditions.

Start of the clinical trial:
Before starting the trial, the approval of the ethical committee need to be available for the Dutch Cancer Society. The trial needs to be started within 12 months after grant submission. You need to communicate the starting date of the trial within a letter to the team of the Dutch Cancer Society.

Evaluation:
When you trial will take longer than 24 months, than you will need to send an evaluation report as mentioned within the general grant conditions. You will need to use the form 'Evaluation form datamanagement clinical trials'.

End of the clinical trial:
Directly after the end of the patient inclusion, you will need to inform the Dutch Cancer Society team within a letter or e-mail. Moreover, within one year after the end of the trial you will provide a report using the form 'report end of clinical trial'.

Datamanagement:
The data management will be subsidized by the Dutch Cancer Society. Datamanagement and trial coordination will be performed by data centers and trial organizations that are affiliated to the Dutch

Cancer Society. You have to notify the trial organizations within the participating regions before the start of the clinical trial together with the planned start date.

Publicity:
Positive publicity including the role of the Dutch Cancer Society is encouraged and motivates the general population to support the cancer society and is of great importance to ensure the financing of clinical trials. It is expected that principal investigators are willing to give input for publicity concerning the trial and the collaboration with the Dutch Cancer Society.

If you have any further questions, please contact department 'doelbesteding' (tel: (020) 570 0450; e-mail: bestedingen@kwf.nl). All correspondence of the project needs to be accompanied with the reference number.

We wish you all the luck with the start of your clinical trial.

With kind regards,

Drs. Michel T. Rudolphie, MBA
General director

Sandra Kloezen
manager doelbesteding

Supplementary files:
- Advice of scientific board
- Information on review process
- Supplementary advice
- General grant conditions Dutch Cancer Society
- Addendum grant conditions clinical trials
- Control protocol Research projects Dutch Cancer Society 12th December 2018
