# Supplementary material for: Radiofrequency ablation and chemotherapy versus chemotherapy alone for locally advanced pancreatic cancer (PELICAN): study protocol for a randomized controlled trial
Source: Trials. 2021 Apr 29;22:313. doi: 10.1186/s13063-021-05248-y (PMC8082784; doi:10.1186/s13063-021-05248-y)
Supplement: Supplementary file 6 — Additional file 6. Translation METC document. [file 13063_2021_5248_MOESM6_ESM.pdf]

To dr. M.G.H. Besselink
Dept of Surgery, G4-196

Amsterdam, 24th December 2014 Medical Ethical Committee
Reference: 2014_325#B20141071
Concerning: positive decision
NL50467.018.14
Pancreatic Locally advanced Irresectable Cancer Ablation in the Netherlands (PELICAN Trial)

Dear dr. Besselink,

The ethical committee of the Amsterdam Medical Center evaluated above-mentioned research file, based upon article 2(2) of the regulation medical scientific research (WMO). We are happy to inform you that our committee

- Competent to judge according to article 2(2a) of WMO
- Working according to ICH-GCP guidelines
- Based upon information as summed up below
- Given article 3, 5 and 6 of WMO
- Establishing that a liability insurance according to article 7(9) WHO was provided

gave a positive decision on this trial and the execution of the trial in the following centers:

Amsterdam Medical Center, University Medical Center Radboud Nijmegen, Isala Clinics Zwolle.

Furthermore, we established that an insurance for the test subjects was taken out by the AMC for subjects included at the AMC, by the Radboud UMC for subjects included at the RadboudUMC and by Isala Clinics for subjects included at the Isala Clinics. All according to the WMO.

<u>Evaluated documents</u>
A1 presenting letter d.d. 6 October 2014
A1 presenting email UMCU d.d. 6 October 2014
A1 presenting letter d.d. 19 December 2014
B1 ABR – form NL50467.018.14 version 02 d.d. 16 December 2014
B2 AMC appendix, signed d.d. 6 October 2014
C1 protocol version 2 d.d. 19 december 2014
E1E2 subject information and informed consent form version 2 d.d. 19 December 2014
G1 Proof WMO insurance Centramed Radboud UMC d.d. 2 October 2014
G1 Proof WMO insurance van Lanschot Chabot Isala Clinics d.d. 17 December 2013
G1 Proof liability insurance Centramed Radboud UMC d.d. December 2012
H1 Curriculum vitae independent doctor Tanis, AMC
H2 Curriculum vitae coordinating investigator drs. Rombouts, UMCU
I2 Research declaration Radboud UMC d.d. 3 October 2014
I2 Research declaration Isala Clinics d.d. 10 November 2014
I3 Curriculum vitae principle investigator van Laarhoven Radboud UMC
I3 Curriculum vitae principle investigator Besselink AMC
I3 Curriculum vitae principle investigator Patijn Isala Clinics
K5 Data safety monitoring board version d.d. 6 October 2014
K6 Data monitoring plan version 1 d.d. 6 October 2014

The complete research file, presented at 6 October 2014 was taken care of and discussed within the meeting of the committee on 23rd of October 2014. The secretary arranged the settlement afterwards and after receiving modifications to the documents, it was decided that this was sufficient to answer the committees questions.

You need to inform the ethical committee the start of the trial, the (prematurely) end of the trial and unexpected adverse events. Furthermore, protocol changes need to be presented and evaluated by the ethical committee. You also need to send us a report of progress every year.

We point out that based upon article 23 of the WMO, those who are directly involved within the decision of the ethical committee, can make notice of appeal within six weeks after the decision of the committee. This notice of appeal needs to be send to the central committee of human research (CCMO), mailbox 16302, 2500 BH Den Haag.

This decision has a term of validity of one year. Within this year the trial needs to be started.

During the evaluation of the trial, the committee consisted of:

Prof. dr. M.P.M. Burger	President, gynaecologist
Ms. Drs. G.H.M. van Ammers	member with the point of view of a study subject
Dr. A.J. Bredenoord	Gastro-enterologist
Dr. M.G.W. Dijkgraaf	Methodologist
Ms. J.M.M. Dijkstra	member with the point of view of a study subject
Dr. M. Figee	Psychiatrist
Prof. dr. R.C.M. Hennekam	Professor in pediatrics and genetics
Prof. dr. J.J. Homan van der Heide	Internal Medicine
Dr. J.M.N.E. Jans	Ethicist
Dr. J.Ph. de Jong	Ethicist
Dr. R.E. Jonkers	Pulmonary doctor / pharmacologist
Prof. dr. A.H.L.C. van Kaan	Pediatrician
Ms dr. E.M. Kemper	Pharmacist / clinical pharmacologist
Dr. M.J.W. Koelemay	Vascular Surgeon
Ms. Dr. S.J. de Kort	Medical Ethicist
Prof. mr. dr. J. Legemaate	Professor Health law
Prof. dr. R.A.A. Malhol	Pharmacist / clinical pharmacologist
Dr. G.A. van Montfrans	Internal Medicine
Ms. Dr. W.M.C. Mulder	Clinical pharmacologist
Dr. P.J. Nederkoorn	Neurologist
Dr. M. Nieuwdorp	Internal Medicine
Ms. Mr.dr. M.C. Ploem	Health Law
Dr. G. ter Riet	Methodologist
Dr. N.W.L. Schep	Trauma surgeon
Prof.dr. A.J.P.M. Smoul	Professor Gastro-enterology
Ms.mr. L.M. Spittuler	member with the point of view of a study subject
Dr. H.L. Tan	Cardiologist
Prof. dr. J.G.P. Tijssen	Professor clinical epidemiology of cardiovascular diseases
Ms. Prof.dr. S. van de Vathorst	Professor palliative care
Prof. dr. M. Vermeulen	Professor neurology
Prof.dr. D.L. Willems	Professor Medical ethics
Prof.dr. A.H. Zwinderman	Professor biostatistics

With kind regards,
The Medical Ethical Committee

Ms. Dr. C.L. van der Wit,
Secretary

Copy: CCMO (central committee of human research)
Copy: AMC medical research BV
Copy: S.J.E. Rombouts
Copy: CRU
